# μ-Cyanido-1:2κ^2^ *N*:*C*-tricyanido-2κ^3^ *C*-(*rac*-5,5,7,12,12,14-hexa­methyl-1,4,8,11-tetra­aza­cyclo­tetra­decane-1κ^4^ *N*,*N*′,*N*′′,*N*′′′)dinickel(II) *N*,*N*-di­methyl­formamide monosolvate hemi­hydrate

**DOI:** 10.1107/S1600536810042625

**Published:** 2010-10-30

**Authors:** Hai-Ming Jiang, Seik Weng Ng

**Affiliations:** a Department of Biology and Chemistry, Hunan University of Science and Engineering, Yongzhou Hunan 425100, People’s Republic of China; b Department of Chemistry, University of Malaya, 50603 Kuala Lumpur, Malaysia

## Abstract

The two Ni^II^ atoms in the title complex, [Ni_2_(CN)_4_(C_16_H_36_N_4_)]·C_3_H_7_NO·0.5H_2_O, are bridged by a cyanide ion. The macrocycle folds around one Ni^II^ atom, which is five-coordinated in an NiN_5_ square-pyramidal geometry. The other Ni^II^ atom is surrounded by the cyanide ions in an NiN_4_ square-planar geometry. The dimethyl­formamide solvent mol­ecule is disordered over two positions in a 0.62 (1):0.38 (1) ratio and the water mol­ecule is disordered about a center of inversion. The dinuclear mol­ecule and solvent mol­ecules are linked by N—H⋯O, N–H⋯N and O—H⋯O hydrogen bonds, forming a three-dimensional network.

## Related literature

For two related structures, see: Jiang *et al.* (2005 ▶, 2007 ▶).
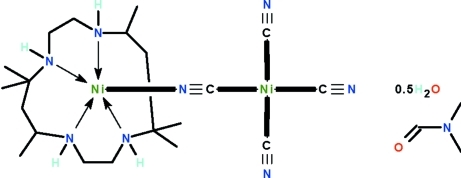


## Experimental

### 

#### Crystal data

[Ni_2_(CN)_4_(C_16_H_36_N_4_)]·C_3_H_7_NO·0.5H_2_O
*M* *_r_* = 588.09
Monoclinic, 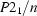

*a* = 10.0122 (5) Å
*b* = 10.2109 (5) Å
*c* = 28.3246 (15) Å
β = 91.468 (1)°
*V* = 2894.8 (3) Å^3^
*Z* = 4
Mo *K*α radiation
μ = 1.33 mm^−1^
*T* = 173 K
0.40 × 0.35 × 0.15 mm

#### Data collection

Bruker SMART APEX diffractometer
Absorption correction: multi-scan (*SADABS*; Sheldrick, 1996 ▶) *T*_min_ = 0.617, *T*_max_ = 0.825
14236 measured reflections
6181 independent reflections
4435 reflections with *I* > 2σ(*I*)
*R*_int_ = 0.035

#### Refinement

*R*[*F* ^2^ > 2σ(*F* ^2^)] = 0.042
*wR*(*F* ^2^) = 0.121
*S* = 1.03
6181 reflections
393 parameters
76 restraints
H atoms treated by a mixture of independent and constrained refinement
Δρ_max_ = 0.55 e Å^−3^
Δρ_min_ = −0.39 e Å^−3^

### 

Data collection: *SMART* (Bruker, 2003 ▶); cell refinement: *SAINT* (Bruker, 2003 ▶); data reduction: *SAINT*; program(s) used to solve structure: *SHELXS97* (Sheldrick, 2008 ▶); program(s) used to refine structure: *SHELXL97* (Sheldrick, 2008 ▶); molecular graphics: *X-SEED* (Barbour, 2001 ▶); software used to prepare material for publication: *publCIF* (Westrip, 2010 ▶).

## Supplementary Material

Crystal structure: contains datablocks global, I. DOI: 10.1107/S1600536810042625/bt5382sup1.cif

Structure factors: contains datablocks I. DOI: 10.1107/S1600536810042625/bt5382Isup2.hkl

Additional supplementary materials:  crystallographic information; 3D view; checkCIF report

## Figures and Tables

**Table 1:** Hydrogen-bond geometry (Å, °)

*D*—H⋯*A*	*D*—H	H⋯*A*	*D*⋯*A*	*D*—H⋯*A*
O1*w*—H11⋯N7	0.84	2.01	2.825 (8)	163
N1—H1⋯N6^i^	0.88 (3)	2.49 (3)	2.854 (4)	105 (3)
N2—H2⋯O1	0.88 (3)	2.46 (2)	3.278 (7)	156 (3)
N2—H2⋯O1′	0.88 (3)	2.05 (2)	2.88 (1)	158 (3)
N3—H3⋯N5	0.87 (3)	2.48 (3)	2.843 (4)	106 (3)
N4—H4⋯O1	0.88 (3)	2.08 (2)	2.927 (8)	162 (3)
N4—H4⋯O1′	0.88 (3)	2.30 (2)	3.14 (1)	160 (3)

Symmetry code: (i) 
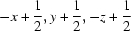
.

**Fig. 1.**
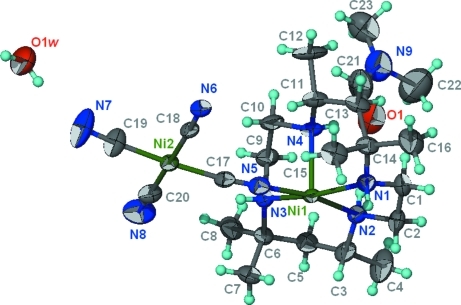
Anisotropic displacement ellipsoid plot (Barbour, 2001) of the title compound at the 70% probability level; hydrogen atoms are shown as spheres of arbitrary radius. The disorder is not shown.

## supplementary crystallographic information

### Comment

We have previously reported the adducts of 5,5,7,12,12,14-hexamethyl-1,4,8,11-tetraazacyclotetradecane with nickel salts. The macrocycle in these adducts chelate to the metal atom in a tetradentate manner. With the counterion as a tetracyanidonickellate(II) dianion, a tetranuclear compound was isolated in which the two dianions each bridges two macrocycle-coordinated nickel atoms (Jiang *et al.*, 2005). Another study reported a macrocycle-nickel–tetracyanidonickellate compound, which exists as a chain (Jiang *et al.*, 2007). In dinuclear [Ni_2_(C_16_H_36_N_4_)]^.^DMF^.^0.5H_2_O (Scheme I, Fig. 1) the two metal atoms are linked by only one cyanide bridge. The dinuclear molecule, DMF molecule and lattice water molecules are linked by N–H···O, N–H···N and O–H···O hydrogen bonds into a linear chain motif.

### Experimental

A DMF solution (20 ml) of dipotassium tetracyanonickellate dihydrate (0.139 g, 0.5 mmol) was layered with an acetonitrile solution (20 ml) of [Ni(*rac*-*L*)](ClO_4_)_2 _(0.272 g, 0.5 mmol) (*rac*-*L* = 5,5,7,12,12,14-hexamethyl-1,4,8,11-tetraazacyclotetradecane) in a glass tube. After about aone month, blue prismatic crystals formed along the walls.

### Refinement

Carbon-bound H-atoms were placed in calculated positions (C–H 0.95–1.00 Å) and were included in the refinement in the riding model approximation, with *U*_iso_(H) set to 1.2–1.5*U*_eq_(C).

The amino H atoms were located in a difference Fourier map, and were refined with a distance restraint of N–H 0.86±0.01 Å and *U*_iso_(H)  = 1.5*U*_eq_(N).

The water molecule is disordered about a center-of-inversion, and is assigned half occupany. Two H atoms were placed on the O atom, with one of them in a chemically sensible position on the basis of hydrogen bonding and *U*_iso_(H)  = 1.5*U*_eq_(O).

The DMF molecule is disordered over two positions in a 62 (1):38 (1) ratio. The carbon–oxygen distances were restrained to 1.25±0.01 Å, the carbon_carbony_l–nitrogen distances to 1.35±0.01 Å and the carbon–carbon_methyl_ distances to 1.45±0.01 Å. The molecule was restrained to lie on a plane. The anisotropic displacement parameters of the disordered atoms were restrained to be nearly isotropic.

### Crystal data

**Table:**

[Ni_2_(CN)_4_(C_16_H_36_N_4_)]·C_3_H_7_NO·0.5H_2_O	*F*(000) = 1252
*M**_r_* = 588.09	*D*_x_ = 1.349 Mg m^−^^3^
Monoclinic, *P*2_1_/*n*	Mo *K*α radiation, λ = 0.71073 Å
Hall symbol: -P 2yn	Cell parameters from 5065 reflections
*a* = 10.0122 (5) Å	θ = 2.5–26.8°
*b* = 10.2109 (5) Å	µ = 1.33 mm^−^^1^
*c* = 28.3246 (15) Å	*T* = 173 K
β = 91.468 (1)°	Prim, blue
*V* = 2894.8 (3) Å^3^	0.40 × 0.35 × 0.15 mm
*Z* = 4	

### Data collection

**Table:**

Bruker SMART APEX diffractometer	6181 independent reflections
Radiation source: fine-focus sealed tube	4435 reflections with *I* > 2σ(*I*)
graphite	*R*_int_ = 0.035
φ and ω scans	θ_max_ = 27.0°, θ_min_ = 2.1°
Absorption correction: multi-scan (*SADABS*; Sheldrick, 1996)	*h* = −12→11
*T*_min_ = 0.617, *T*_max_ = 0.825	*k* = −13→12
14236 measured reflections	*l* = −30→36

### Refinement

**Table:**

Refinement on *F*^2^	Primary atom site location: structure-invariant direct methods
Least-squares matrix: full	Secondary atom site location: difference Fourier map
*R*[*F*^2^ > 2σ(*F*^2^)] = 0.042	Hydrogen site location: inferred from neighbouring sites
*wR*(*F*^2^) = 0.121	H atoms treated by a mixture of independent and constrained refinement
*S* = 1.03	*w* = 1/[σ^2^(*F*_o_^2^) + (0.0666*P*)^2^ + 0.7079*P*] where *P* = (*F*_o_^2^ + 2*F*_c_^2^)/3
6181 reflections	(Δ/σ)_max_ = 0.001
393 parameters	Δρ_max_ = 0.55 e Å^−^^3^
76 restraints	Δρ_min_ = −0.39 e Å^−^^3^

### Fractional atomic coordinates and isotropic or equivalent isotropic displacement parameters (Å^2^)

**Table:**

	*x*	*y*	*z*	*U*_iso_*/*U*_eq_	Occ. (<1)
Ni1	0.40876 (4)	0.41556 (4)	0.163713 (13)	0.02095 (12)	
Ni2	0.41778 (4)	0.28354 (4)	0.332984 (14)	0.02452 (13)	
O1W	0.5942 (8)	−0.0422 (7)	0.4816 (2)	0.076 (2)	0.50
H11	0.5490	0.0200	0.4702	0.115*	0.50
H12	0.5602	−0.0657	0.5071	0.115*	0.50
N1	0.2327 (3)	0.3662 (3)	0.12200 (10)	0.0284 (6)	
H1	0.177 (3)	0.429 (3)	0.1287 (12)	0.034*	
N2	0.4790 (3)	0.4921 (3)	0.09926 (10)	0.0282 (6)	
H2	0.532 (3)	0.433 (3)	0.0872 (11)	0.034*	
N3	0.6097 (3)	0.4408 (3)	0.19194 (9)	0.0261 (6)	
H3	0.599 (3)	0.435 (3)	0.2224 (4)	0.031*	
N4	0.4697 (3)	0.2182 (3)	0.15349 (11)	0.0285 (6)	
H4	0.491 (4)	0.213 (4)	0.1236 (5)	0.034*	
N5	0.3642 (3)	0.3681 (3)	0.23283 (10)	0.0310 (6)	
N6	0.1889 (3)	0.0938 (3)	0.32576 (10)	0.0289 (6)	
N7	0.4806 (4)	0.1648 (5)	0.42804 (14)	0.0729 (13)	
N8	0.6614 (4)	0.4584 (4)	0.33648 (15)	0.0586 (10)	
C1	0.2720 (4)	0.3856 (4)	0.07273 (12)	0.0358 (8)	
H1A	0.1911	0.3951	0.0522	0.043*	
H1B	0.3221	0.3080	0.0619	0.043*	
C2	0.3578 (3)	0.5057 (4)	0.06859 (12)	0.0335 (8)	
H2A	0.3839	0.5174	0.0354	0.040*	
H2B	0.3067	0.5840	0.0781	0.040*	
C3	0.5572 (4)	0.6160 (4)	0.10311 (13)	0.0360 (8)	
H3A	0.5026	0.6822	0.1201	0.043*	
C4	0.5928 (5)	0.6725 (5)	0.05472 (15)	0.0663 (15)	
H4A	0.5106	0.6910	0.0364	0.100*	
H4B	0.6468	0.6089	0.0377	0.100*	
H4C	0.6437	0.7538	0.0592	0.100*	
C5	0.6883 (4)	0.5960 (4)	0.13084 (12)	0.0341 (8)	
H5A	0.7422	0.6765	0.1273	0.041*	
H5B	0.7373	0.5242	0.1154	0.041*	
C6	0.6846 (3)	0.5644 (3)	0.18365 (12)	0.0288 (7)	
C7	0.6187 (4)	0.6735 (3)	0.21114 (13)	0.0370 (8)	
H7A	0.5250	0.6817	0.2007	0.056*	
H7B	0.6653	0.7562	0.2055	0.056*	
H7C	0.6234	0.6530	0.2449	0.056*	
C8	0.8294 (4)	0.5519 (4)	0.20212 (14)	0.0418 (9)	
H8A	0.8302	0.5166	0.2343	0.063*	
H8B	0.8718	0.6384	0.2023	0.063*	
H8C	0.8785	0.4928	0.1816	0.063*	
C9	0.6805 (3)	0.3219 (3)	0.17783 (14)	0.0347 (8)	
H9A	0.7628	0.3116	0.1976	0.042*	
H9B	0.7068	0.3298	0.1445	0.042*	
C10	0.5924 (4)	0.2031 (3)	0.18337 (14)	0.0366 (9)	
H10A	0.6413	0.1234	0.1739	0.044*	
H10B	0.5682	0.1933	0.2169	0.044*	
C11	0.3705 (4)	0.1138 (3)	0.16224 (14)	0.0356 (8)	
H11A	0.3366	0.1257	0.1949	0.043*	
C12	0.4307 (4)	−0.0238 (4)	0.15890 (18)	0.0563 (12)	
H12A	0.5060	−0.0319	0.1815	0.084*	
H12B	0.4622	−0.0385	0.1268	0.084*	
H12C	0.3625	−0.0891	0.1662	0.084*	
C13	0.2526 (4)	0.1236 (3)	0.12764 (13)	0.0359 (8)	
H13A	0.1985	0.0431	0.1311	0.043*	
H13B	0.2884	0.1224	0.0954	0.043*	
C14	0.1569 (3)	0.2413 (3)	0.13060 (13)	0.0322 (8)	
C15	0.0965 (4)	0.2505 (4)	0.17929 (14)	0.0407 (9)	
H15A	0.0348	0.3249	0.1801	0.061*	
H15B	0.1679	0.2629	0.2032	0.061*	
H15C	0.0479	0.1695	0.1860	0.061*	
C16	0.0434 (4)	0.2243 (4)	0.09397 (15)	0.0504 (11)	
H16A	−0.0098	0.3048	0.0922	0.076*	
H16B	−0.0137	0.1511	0.1032	0.076*	
H16C	0.0809	0.2060	0.0630	0.076*	
C17	0.3803 (3)	0.3420 (3)	0.27219 (12)	0.0266 (7)	
C18	0.2738 (3)	0.1684 (3)	0.32772 (11)	0.0253 (7)	
C19	0.4574 (4)	0.2135 (4)	0.39238 (14)	0.0441 (10)	
C20	0.5677 (4)	0.3942 (4)	0.33603 (13)	0.0354 (8)	
O1	0.5992 (7)	0.2131 (7)	0.0620 (3)	0.064 (2)	0.620 (6)
N9	0.7506 (6)	0.0709 (6)	0.0306 (2)	0.063 (2)	0.620 (6)
C21	0.6732 (6)	0.1167 (7)	0.0637 (2)	0.058 (2)	0.620 (6)
H21	0.6746	0.0693	0.0926	0.070*	0.620 (6)
C22	0.7521 (12)	0.1403 (11)	−0.0141 (3)	0.100 (4)	0.620 (6)
H22A	0.6744	0.1984	−0.0166	0.150*	0.620 (6)
H22B	0.7489	0.0770	−0.0401	0.150*	0.620 (6)
H22C	0.8341	0.1923	−0.0157	0.150*	0.620 (6)
C23	0.8352 (7)	−0.0424 (7)	0.0360 (3)	0.064 (2)	0.620 (6)
H23A	0.8188	−0.0843	0.0664	0.095*	0.620 (6)
H23B	0.9290	−0.0156	0.0348	0.095*	0.620 (6)
H23C	0.8152	−0.1046	0.0104	0.095*	0.620 (6)
O1'	0.5863 (12)	0.2654 (13)	0.0530 (4)	0.063 (4)	0.380 (6)
N9'	0.7086 (10)	0.1118 (10)	0.0163 (2)	0.053 (3)	0.380 (6)
C21'	0.6906 (10)	0.2298 (11)	0.0351 (3)	0.044 (3)	0.380 (6)
H21B	0.7628	0.2902	0.0346	0.053*	0.380 (6)
C22'	0.6064 (16)	0.0150 (16)	0.0151 (7)	0.120 (7)	0.380 (6)
H22D	0.5189	0.0578	0.0126	0.180*	0.380 (6)
H22E	0.6118	−0.0371	0.0442	0.180*	0.380 (6)
H22F	0.6182	−0.0423	−0.0122	0.180*	0.380 (6)
C23'	0.8349 (13)	0.0747 (18)	−0.0046 (5)	0.095 (6)	0.380 (6)
H23D	0.9048	0.1366	0.0055	0.142*	0.380 (6)
H23E	0.8250	0.0763	−0.0391	0.142*	0.380 (6)
H23F	0.8598	−0.0138	0.0058	0.142*	0.380 (6)

### Atomic displacement parameters (Å^2^)

**Table:**

	*U*^11^	*U*^22^	*U*^33^	*U*^12^	*U*^13^	*U*^23^
Ni1	0.0237 (2)	0.0160 (2)	0.0233 (2)	0.00309 (15)	0.00128 (15)	0.00060 (16)
Ni2	0.0249 (2)	0.0212 (2)	0.0276 (2)	−0.00409 (16)	0.00209 (17)	0.00290 (17)
O1W	0.118 (6)	0.062 (4)	0.048 (4)	0.007 (4)	−0.008 (4)	0.012 (3)
N1	0.0267 (15)	0.0263 (15)	0.0323 (15)	0.0035 (11)	−0.0017 (12)	−0.0028 (12)
N2	0.0302 (16)	0.0263 (15)	0.0281 (15)	0.0010 (12)	0.0012 (12)	0.0024 (12)
N3	0.0300 (15)	0.0219 (14)	0.0262 (14)	0.0001 (11)	−0.0007 (12)	0.0023 (12)
N4	0.0296 (15)	0.0164 (14)	0.0395 (16)	0.0021 (11)	0.0032 (13)	0.0003 (12)
N5	0.0344 (16)	0.0250 (15)	0.0338 (17)	−0.0053 (12)	0.0064 (13)	0.0025 (13)
N6	0.0306 (16)	0.0225 (15)	0.0336 (16)	−0.0002 (12)	0.0029 (12)	0.0012 (12)
N7	0.054 (3)	0.112 (4)	0.053 (2)	−0.006 (2)	−0.0085 (19)	0.040 (3)
N8	0.041 (2)	0.044 (2)	0.091 (3)	−0.0159 (17)	0.0035 (19)	0.000 (2)
C1	0.040 (2)	0.040 (2)	0.0273 (18)	−0.0005 (16)	−0.0068 (15)	−0.0015 (16)
C2	0.038 (2)	0.040 (2)	0.0219 (17)	0.0006 (16)	−0.0022 (15)	0.0066 (15)
C3	0.042 (2)	0.031 (2)	0.0351 (19)	−0.0068 (16)	0.0025 (16)	0.0113 (16)
C4	0.074 (3)	0.078 (4)	0.046 (3)	−0.032 (3)	−0.007 (2)	0.031 (3)
C5	0.036 (2)	0.0317 (19)	0.0353 (19)	−0.0061 (15)	0.0055 (15)	0.0002 (16)
C6	0.0314 (19)	0.0251 (18)	0.0297 (18)	−0.0053 (14)	−0.0007 (14)	0.0002 (14)
C7	0.041 (2)	0.0260 (18)	0.044 (2)	−0.0066 (16)	0.0026 (17)	−0.0060 (16)
C8	0.036 (2)	0.041 (2)	0.048 (2)	−0.0102 (17)	−0.0057 (17)	−0.0059 (18)
C9	0.0248 (18)	0.0279 (19)	0.051 (2)	0.0058 (14)	−0.0082 (16)	−0.0004 (16)
C10	0.038 (2)	0.0198 (18)	0.052 (2)	0.0080 (14)	−0.0071 (17)	0.0012 (16)
C11	0.035 (2)	0.0197 (17)	0.052 (2)	0.0012 (14)	0.0032 (17)	−0.0002 (16)
C12	0.050 (3)	0.0140 (19)	0.105 (4)	0.0030 (17)	0.004 (2)	0.003 (2)
C13	0.035 (2)	0.0240 (18)	0.049 (2)	−0.0072 (15)	0.0056 (17)	−0.0084 (16)
C14	0.0290 (19)	0.0250 (18)	0.042 (2)	−0.0057 (14)	0.0011 (15)	−0.0026 (15)
C15	0.0267 (19)	0.042 (2)	0.054 (2)	−0.0040 (16)	0.0095 (17)	−0.0040 (18)
C16	0.037 (2)	0.054 (3)	0.060 (3)	−0.0094 (19)	−0.0093 (19)	−0.009 (2)
C17	0.0288 (18)	0.0190 (16)	0.0322 (19)	−0.0051 (13)	0.0041 (14)	0.0025 (14)
C18	0.0289 (18)	0.0194 (16)	0.0277 (17)	−0.0032 (14)	0.0011 (14)	0.0053 (13)
C19	0.031 (2)	0.060 (3)	0.042 (2)	−0.0073 (18)	−0.0011 (17)	0.015 (2)
C20	0.034 (2)	0.0273 (19)	0.045 (2)	−0.0002 (15)	0.0024 (16)	0.0038 (16)
O1	0.060 (4)	0.084 (6)	0.049 (4)	0.033 (4)	0.013 (3)	−0.003 (4)
N9	0.060 (5)	0.079 (5)	0.050 (4)	0.013 (4)	0.011 (3)	−0.003 (4)
C21	0.057 (4)	0.080 (5)	0.038 (4)	0.008 (4)	0.008 (3)	−0.007 (4)
C22	0.123 (7)	0.097 (7)	0.080 (6)	0.030 (6)	0.010 (6)	0.015 (5)
C23	0.054 (4)	0.074 (5)	0.064 (5)	−0.004 (4)	0.023 (4)	−0.015 (4)
O1'	0.068 (6)	0.065 (7)	0.057 (6)	0.032 (5)	0.002 (5)	−0.017 (5)
N9'	0.052 (6)	0.053 (6)	0.056 (7)	0.000 (5)	0.018 (5)	−0.023 (5)
C21'	0.047 (6)	0.047 (6)	0.037 (5)	0.005 (5)	−0.002 (4)	−0.004 (5)
C22'	0.113 (10)	0.091 (9)	0.156 (11)	−0.002 (8)	0.022 (8)	−0.014 (8)
C23'	0.084 (8)	0.105 (9)	0.095 (9)	0.017 (7)	0.008 (7)	−0.036 (8)

### Geometric parameters (Å, °)

**Table:**

Ni1—N5	2.076 (3)	C8—H8A	0.9800
Ni1—N6^i^	2.091 (3)	C8—H8B	0.9800
Ni1—N2	2.122 (3)	C8—H8C	0.9800
Ni1—N4	2.127 (3)	C9—C10	1.510 (5)
Ni1—N1	2.156 (3)	C9—H9A	0.9900
Ni1—N3	2.161 (3)	C9—H9B	0.9900
Ni2—C17	1.852 (3)	C10—H10A	0.9900
Ni2—C19	1.861 (4)	C10—H10B	0.9900
Ni2—C18	1.863 (3)	C11—C13	1.519 (5)
Ni2—C20	1.879 (4)	C11—C12	1.533 (5)
O1W—H11	0.84	C11—H11A	1.0000
O1W—H12	0.84	C12—H12A	0.9800
N1—C1	1.473 (4)	C12—H12B	0.9800
N1—C14	1.507 (4)	C12—H12C	0.9800
N1—H1	0.88 (3)	C13—C14	1.540 (5)
N2—C2	1.481 (4)	C13—H13A	0.9900
N2—C3	1.490 (4)	C13—H13B	0.9900
N2—H2	0.88 (3)	C14—C15	1.523 (5)
N3—C9	1.467 (4)	C14—C16	1.529 (5)
N3—C6	1.489 (4)	C15—H15A	0.9800
N3—H3	0.87 (3)	C15—H15B	0.9800
N4—C10	1.482 (4)	C15—H15C	0.9800
N4—C11	1.483 (4)	C16—H16A	0.9800
N4—H4	0.88 (3)	C16—H16B	0.9800
N5—C17	1.154 (4)	C16—H16C	0.9800
N6—C18	1.142 (4)	O1—C21	1.233 (7)
N6—Ni1^ii^	2.091 (3)	N9—C21	1.318 (7)
N7—C19	1.144 (5)	N9—C23	1.440 (7)
N8—C20	1.144 (5)	N9—C22	1.450 (8)
C1—C2	1.504 (5)	C21—H21	0.9500
C1—H1A	0.9900	C22—H22A	0.9800
C1—H1B	0.9900	C22—H22B	0.9800
C2—H2A	0.9900	C22—H22C	0.9800
C2—H2B	0.9900	C23—H23A	0.9800
C3—C5	1.526 (5)	C23—H23B	0.9800
C3—C4	1.537 (5)	C23—H23C	0.9800
C3—H3A	1.0000	O1'—C21'	1.228 (9)
C4—H4A	0.9800	N9'—C21'	1.331 (14)
C4—H4B	0.9800	N9'—C22'	1.422 (9)
C4—H4C	0.9800	N9'—C23'	1.461 (9)
C5—C6	1.532 (5)	C21'—H21B	0.9500
C5—H5A	0.9900	C22'—H22D	0.9800
C5—H5B	0.9900	C22'—H22E	0.9800
C6—C7	1.519 (5)	C22'—H22F	0.9800
C6—C8	1.534 (5)	C23'—H23D	0.9800
C7—H7A	0.9800	C23'—H23E	0.9800
C7—H7B	0.9800	C23'—H23F	0.9800
C7—H7C	0.9800		
			
N5—Ni1—N6^i^	87.47 (11)	H7A—C7—H7B	109.5
N5—Ni1—N2	168.80 (11)	C6—C7—H7C	109.5
N6^i^—Ni1—N2	88.20 (11)	H7A—C7—H7C	109.5
N5—Ni1—N4	88.68 (11)	H7B—C7—H7C	109.5
N6^i^—Ni1—N4	168.73 (11)	C6—C8—H8A	109.5
N2—Ni1—N4	97.42 (11)	C6—C8—H8B	109.5
N5—Ni1—N1	105.64 (11)	H8A—C8—H8B	109.5
N6^i^—Ni1—N1	84.45 (11)	C6—C8—H8C	109.5
N2—Ni1—N1	84.20 (11)	H8A—C8—H8C	109.5
N4—Ni1—N1	86.41 (11)	H8B—C8—H8C	109.5
N5—Ni1—N3	84.24 (11)	N3—C9—C10	110.4 (3)
N6^i^—Ni1—N3	106.11 (11)	N3—C9—H9A	109.6
N2—Ni1—N3	87.05 (11)	C10—C9—H9A	109.6
N4—Ni1—N3	84.01 (11)	N3—C9—H9B	109.6
N1—Ni1—N3	166.08 (10)	C10—C9—H9B	109.6
C17—Ni2—C19	176.11 (17)	H9A—C9—H9B	108.1
C17—Ni2—C18	89.49 (14)	N4—C10—C9	109.5 (3)
C19—Ni2—C18	88.66 (15)	N4—C10—H10A	109.8
C17—Ni2—C20	89.51 (15)	C9—C10—H10A	109.8
C19—Ni2—C20	92.17 (16)	N4—C10—H10B	109.8
C18—Ni2—C20	177.02 (15)	C9—C10—H10B	109.8
H11—O1W—H12	108.8	H10A—C10—H10B	108.2
C1—N1—C14	114.5 (3)	N4—C11—C13	111.0 (3)
C1—N1—Ni1	104.7 (2)	N4—C11—C12	112.5 (3)
C14—N1—Ni1	121.1 (2)	C13—C11—C12	108.7 (3)
C1—N1—H1	107 (2)	N4—C11—H11A	108.2
C14—N1—H1	105 (2)	C13—C11—H11A	108.2
Ni1—N1—H1	103 (2)	C12—C11—H11A	108.2
C2—N2—C3	112.6 (3)	C11—C12—H12A	109.5
C2—N2—Ni1	104.8 (2)	C11—C12—H12B	109.5
C3—N2—Ni1	115.8 (2)	H12A—C12—H12B	109.5
C2—N2—H2	109 (2)	C11—C12—H12C	109.5
C3—N2—H2	107 (2)	H12A—C12—H12C	109.5
Ni1—N2—H2	107 (2)	H12B—C12—H12C	109.5
C9—N3—C6	114.2 (3)	C11—C13—C14	119.4 (3)
C9—N3—Ni1	104.6 (2)	C11—C13—H13A	107.5
C6—N3—Ni1	120.7 (2)	C14—C13—H13A	107.5
C9—N3—H3	106 (2)	C11—C13—H13B	107.5
C6—N3—H3	107 (2)	C14—C13—H13B	107.5
Ni1—N3—H3	103 (2)	H13A—C13—H13B	107.0
C10—N4—C11	112.3 (3)	N1—C14—C15	107.9 (3)
C10—N4—Ni1	104.9 (2)	N1—C14—C16	110.8 (3)
C11—N4—Ni1	117.6 (2)	C15—C14—C16	108.4 (3)
C10—N4—H4	109 (2)	N1—C14—C13	109.6 (3)
C11—N4—H4	107 (2)	C15—C14—C13	111.1 (3)
Ni1—N4—H4	105 (2)	C16—C14—C13	109.1 (3)
C17—N5—Ni1	159.5 (3)	C14—C15—H15A	109.5
C18—N6—Ni1^ii^	157.5 (3)	C14—C15—H15B	109.5
N1—C1—C2	110.5 (3)	H15A—C15—H15B	109.5
N1—C1—H1A	109.5	C14—C15—H15C	109.5
C2—C1—H1A	109.5	H15A—C15—H15C	109.5
N1—C1—H1B	109.5	H15B—C15—H15C	109.5
C2—C1—H1B	109.5	C14—C16—H16A	109.5
H1A—C1—H1B	108.1	C14—C16—H16B	109.5
N2—C2—C1	109.8 (3)	H16A—C16—H16B	109.5
N2—C2—H2A	109.7	C14—C16—H16C	109.5
C1—C2—H2A	109.7	H16A—C16—H16C	109.5
N2—C2—H2B	109.7	H16B—C16—H16C	109.5
C1—C2—H2B	109.7	N5—C17—Ni2	173.3 (3)
H2A—C2—H2B	108.2	N6—C18—Ni2	176.8 (3)
N2—C3—C5	111.6 (3)	N7—C19—Ni2	176.8 (4)
N2—C3—C4	112.8 (3)	N8—C20—Ni2	177.1 (4)
C5—C3—C4	107.0 (3)	C21—N9—C23	124.4 (7)
N2—C3—H3A	108.5	C21—N9—C22	117.8 (7)
C5—C3—H3A	108.5	C23—N9—C22	117.8 (7)
C4—C3—H3A	108.5	O1—C21—N9	128.3 (7)
C3—C4—H4A	109.5	O1—C21—H21	115.8
C3—C4—H4B	109.5	N9—C21—H21	115.8
H4A—C4—H4B	109.5	C21'—N9'—C22'	122.3 (11)
C3—C4—H4C	109.5	C21'—N9'—C23'	121.6 (11)
H4A—C4—H4C	109.5	C22'—N9'—C23'	116.1 (13)
H4B—C4—H4C	109.5	O1'—C21'—N9'	123.9 (12)
C3—C5—C6	119.3 (3)	O1'—C21'—H21B	118.0
C3—C5—H5A	107.5	N9'—C21'—H21B	118.0
C6—C5—H5A	107.5	N9'—C22'—H22D	109.5
C3—C5—H5B	107.5	N9'—C22'—H22E	109.5
C6—C5—H5B	107.5	H22D—C22'—H22E	109.5
H5A—C5—H5B	107.0	N9'—C22'—H22F	109.5
N3—C6—C7	108.3 (3)	H22D—C22'—H22F	109.5
N3—C6—C5	110.9 (3)	H22E—C22'—H22F	109.5
C7—C6—C5	111.6 (3)	N9'—C23'—H23D	109.5
N3—C6—C8	110.6 (3)	N9'—C23'—H23E	109.5
C7—C6—C8	107.8 (3)	H23D—C23'—H23E	109.5
C5—C6—C8	107.7 (3)	N9'—C23'—H23F	109.5
C6—C7—H7A	109.5	H23D—C23'—H23F	109.5
C6—C7—H7B	109.5	H23E—C23'—H23F	109.5
			
N5—Ni1—N1—C1	−173.4 (2)	C14—N1—C1—C2	−174.7 (3)
N6^i^—Ni1—N1—C1	100.8 (2)	Ni1—N1—C1—C2	−39.7 (3)
N2—Ni1—N1—C1	12.0 (2)	C3—N2—C2—C1	−170.5 (3)
N4—Ni1—N1—C1	−85.8 (2)	Ni1—N2—C2—C1	−43.8 (3)
N3—Ni1—N1—C1	−39.3 (5)	N1—C1—C2—N2	59.1 (4)
N5—Ni1—N1—C14	−42.2 (3)	C2—N2—C3—C5	−174.4 (3)
N6^i^—Ni1—N1—C14	−128.0 (2)	Ni1—N2—C3—C5	65.1 (3)
N2—Ni1—N1—C14	143.3 (2)	C2—N2—C3—C4	−53.9 (4)
N4—Ni1—N1—C14	45.4 (2)	Ni1—N2—C3—C4	−174.4 (3)
N3—Ni1—N1—C14	92.0 (5)	N2—C3—C5—C6	−67.3 (4)
N5—Ni1—N2—C2	−134.8 (5)	C4—C3—C5—C6	168.9 (3)
N6^i^—Ni1—N2—C2	−67.6 (2)	C9—N3—C6—C7	−165.2 (3)
N4—Ni1—N2—C2	102.6 (2)	Ni1—N3—C6—C7	68.8 (3)
N1—Ni1—N2—C2	17.0 (2)	C9—N3—C6—C5	72.1 (4)
N3—Ni1—N2—C2	−173.8 (2)	Ni1—N3—C6—C5	−53.9 (3)
N5—Ni1—N2—C3	−10.2 (7)	C9—N3—C6—C8	−47.3 (4)
N6^i^—Ni1—N2—C3	57.1 (2)	Ni1—N3—C6—C8	−173.4 (2)
N4—Ni1—N2—C3	−132.7 (2)	C3—C5—C6—N3	59.8 (4)
N1—Ni1—N2—C3	141.7 (2)	C3—C5—C6—C7	−61.0 (4)
N3—Ni1—N2—C3	−49.1 (2)	C3—C5—C6—C8	−179.1 (3)
N5—Ni1—N3—C9	102.4 (2)	C6—N3—C9—C10	−174.9 (3)
N6^i^—Ni1—N3—C9	−171.9 (2)	Ni1—N3—C9—C10	−40.9 (3)
N2—Ni1—N3—C9	−84.7 (2)	C11—N4—C10—C9	−172.1 (3)
N4—Ni1—N3—C9	13.1 (2)	Ni1—N4—C10—C9	−43.3 (3)
N1—Ni1—N3—C9	−33.6 (5)	N3—C9—C10—N4	59.6 (4)
N5—Ni1—N3—C6	−127.3 (2)	C10—N4—C11—C13	−174.0 (3)
N6^i^—Ni1—N3—C6	−41.6 (2)	Ni1—N4—C11—C13	64.1 (3)
N2—Ni1—N3—C6	45.6 (2)	C10—N4—C11—C12	−52.0 (4)
N4—Ni1—N3—C6	143.4 (2)	Ni1—N4—C11—C12	−173.8 (3)
N1—Ni1—N3—C6	96.7 (5)	N4—C11—C13—C14	−67.8 (4)
N5—Ni1—N4—C10	−68.0 (2)	C12—C11—C13—C14	168.0 (3)
N6^i^—Ni1—N4—C10	−138.0 (5)	C1—N1—C14—C15	−167.1 (3)
N2—Ni1—N4—C10	102.6 (2)	Ni1—N1—C14—C15	66.0 (3)
N1—Ni1—N4—C10	−173.8 (2)	C1—N1—C14—C16	−48.6 (4)
N3—Ni1—N4—C10	16.3 (2)	Ni1—N1—C14—C16	−175.5 (2)
N5—Ni1—N4—C11	57.5 (3)	C1—N1—C14—C13	71.8 (4)
N6^i^—Ni1—N4—C11	−12.5 (7)	Ni1—N1—C14—C13	−55.1 (3)
N2—Ni1—N4—C11	−131.9 (3)	C11—C13—C14—N1	61.6 (4)
N1—Ni1—N4—C11	−48.2 (3)	C11—C13—C14—C15	−57.6 (4)
N3—Ni1—N4—C11	141.9 (3)	C11—C13—C14—C16	−177.0 (3)
N6^i^—Ni1—N5—C17	−117.1 (8)	C23—N9—C21—O1	179.4 (4)
N2—Ni1—N5—C17	−49.8 (11)	C22—N9—C21—O1	0.2 (4)
N4—Ni1—N5—C17	73.5 (8)	C22'—N9'—C21'—O1'	0.4 (4)
N1—Ni1—N5—C17	159.3 (8)	C23'—N9'—C21'—O1'	180.0 (3)
N3—Ni1—N5—C17	−10.7 (8)		

Symmetry codes: (i) −*x*+1/2, *y*+1/2, −*z*+1/2; (ii) −*x*+1/2, *y*−1/2, −*z*+1/2.

### Hydrogen-bond geometry (Å, °)

**Table:**

*D*—H···*A*	*D*—H	H···*A*	*D*···*A*	*D*—H···*A*
O1w—H11···N7	0.84	2.01	2.825 (8)	163
N1—H1···N6^i^	0.88 (3)	2.49 (3)	2.854 (4)	105 (3)
N2—H2···O1	0.88 (3)	2.46 (2)	3.278 (7)	156 (3)
N2—H2···O1'	0.88 (3)	2.05 (2)	2.88 (1)	158 (3)
N3—H3···N5	0.87 (3)	2.48 (3)	2.843 (4)	106 (3)
N4—H4···O1	0.88 (3)	2.08 (2)	2.927 (8)	162 (3)
N4—H4···O1'	0.88 (3)	2.30 (2)	3.14 (1)	160 (3)

Symmetry codes: (i) −*x*+1/2, *y*+1/2, −*z*+1/2.
